# Comparative Study of The Influence of EDTA and Sodium Heparin on Long Term Storage of Cattle DNA

**DOI:** 10.22074/cellj.2015.526

**Published:** 2015-04-08

**Authors:** Rosaiah Kotikalapudi, Rajesh K. Patel

**Affiliations:** 1 Department of Animal Biotechnology, Sandor Life Sciences Pvt. Ltd., Hyderabad, India; 2 Department of Animal Biotechnology, Sandor Animal Biogenics Pvt. Ltd., Hyderabad, India

**Keywords:** Heparin, EDTA, Cattle, DNA Stability

## Abstract

Blood collection in heparin tubes for cytogenetic, and ethylenediaminetetraacetic acid (EDTA) tubes for molecular genetics applications respectively, are routine practices everywhere. If blood samples are required for cytogenetics as well as DNA work, two samples from each animal are usually collected, which leads to wastage of time and money. The present study tried to explore the possibilities of collecting a single blood sample in a heparinised tube for use in both applications. Two blood samples were collected from the same animals; one in a heparin tube and the other in an EDTA tube. DNA was extracted and stored at the same temperature and for the same durations. Comparative studies revealed that the DNA samples extracted from blood using these two different coagulants give more or less the same quality of results especially for polymerase chain reaction (PCR) based applications in cattle. The purpose of the present study was to establish the possibility of using heparin blood for chromosomal studies as well as for molecular biology. Such a practice will obviously save time and money in collecting samples in duplicate.

Long term storage of DNA samples is required to create a genomic DNA bank for cattle and to minimize the future cost of research. Successful long term storage depends on the stability of the DNA samples which in turn depends on storage conditions. DNA is an inherently stable molecule frequently used in molecular research. Research scientists utilizing blood in their studies have unique needs depending on their downstream applications. Blood can be collected using different anticoagulants including citrate, ethylenediaminetetraacetic acid (EDTA) or heparin. The type of anticoagulant used in blood collection can affect the results from blood DNA isolation, and may influence the results of research-based or diagnostic tests associated with blood. Research scientists must ensure that their blood DNA isolation method is flexible, i.e. it can work efficiently in isolating DNA from the specific anticoagulant used for the blood collection. EDTA is the anticoagulant of choice for blood collection for DNA extractions because it inhibits DNase activity and does not change the quantity of DNA. However, it does affect magnesium concentrations in downstream applications. Heparin should be avoided, as it can bind to DNA during purification and can inhibit Taq polymerase used for polymerase chain reaction (PCR) (1). In other words, sodium heparin, an anticoagulant used widely for blood collection, has been known to inhibit DNA polymerase activity in PCR assays (2). Irrespective of the anticoagulant, the vacutainer tube should be inverted several times to mix the blood. Blood can be shipped at ambient temperature, but if the delay between collection and extraction is more than three days, there will be some degradation of DNA and the yield will be lower than that from fresh blood. On the whole, it is advisable to transport blood samples at 4˚C to avoid degradation of biological samples. The most common method of storage of DNA is as a suspension in ethanol at -80˚C, however, isolated DNA can be stored at 4˚C for several weeks, at -20˚C for several months and at -80˚C for several years. Factors that affect the stability of biological samples include anticoagulants (3), stabilizing agents (4), temperature (5), timing before initial processing (6), sterility, endogenous degrading properties (enzymes, cell death), etc., (7). Nuclease contamination must be avoided but the main threat to DNA preservation is usually chemical degradation. The concentration of magnesium ion in the buffers is critical to obtain intact and, high molecular weight DNA. It has been shown that DNA samples remain intact for longer when DNA is dissolved in higher concentrations of EDTA (2). 

The aim of present study to find out I. the quality and quantity of DNA isolated from blood collected in EDTA and sodium heparin vacutainer tubes, II. the effect of EDTA and sodium heparin on DNA during long term storage at -20˚C and III. effects on the application of such DNA for molecular techniques.

Two ml of blood were randomly collected in 4 ml EDTA tubes (ref 367861) and in heparin tubes (ref 367871) (BD Vacutainer, USA) from the same 10 healthy Holstein bulls, by a trained veterinarian. Blood samples were transported in cool packs from the farm to our laboratory. As soon as the blood samples reached to the laboratory, they were kept refrigerated at 4˚C. DNA was extracted within 5 days of collection using the phenol-chloroform (SRL, India) standard protocol, with little modification of the procedure. After extraction, the quality and quantity of DNA extracted from EDTA and heparinised blood were determined using nano-spectrophotometry (Thermo Fisher, USA) at 260/280 optical density (OD) as indicated in table 1. The experiment was designed in such a manner that this procedure was repeated after every three months of storage at -20˚C. The quality and quantity of DNA were recorded every time and differences between the first and second observations were calculated. In the same way, differences in the quality and quantity of DNA from both types of extraction were calculated between third and second reading, fourth and third reading, and the fifth and fourth reading as shown in table 1. Finally the quality of DNA after long term storage was visualized on 0.8% agarose gel (Lonza, USA) electrophoresis and subjected to PCR. As described earlier (3) with minor modifications, the PCR was set up containing 100 ng of genomic DNA template, 0.4 pM each of forward (5ˊCCCACTGGCTAGGAATCGTT3ˊ) and reverse (5ˊCAAGGCAATGTCATATCCAC3ˊ) primers, 1X PCR buffer, 400 μM each deoxynucleotide triphosphates (dNTP) and 1 U of Taq DNA polymerase, in a final reaction volume of 25 μl. The PCR buffer, dNTP and Taq DNA polymerase used in PCR are from Kapa Biosystem, USA, whereas primers are from MWG-Biotech from Germany. The PCR was carried out using a thermal cycler (Applied Bio System, USA). Initial denaturation was achieved at 94˚C for 3 minutes followed by 30 cycles of 94˚C for 1.5 minutes, annealing of primers at 55˚C for 1 minute and extension at 72˚C for 2 minutes followed by final extension at 72˚C for 10 minutes. The amplification products were analyzed in 1.5% agarose gels, stained with ethidium bromide (Sigma, USA) and viewed under ultra violate (UV) light.

In order to determine the yield of DNA isolated from each blood sample collected using the different anticoagulants, samples were measured using the nano-spectrophotometric method. The highest DNA yield was obtained from blood collected on EDTA (853.1 μg). Table 1 shows the DNA concentration and reading taken at 260/280 OD on 23.10.12 for both samples. The first DNA concentration readings ranged from 29.3 to 432.8 and from 37.7 to 853.1 for heparin and EDTA respectively. The first OD reading ranged from 1.83 to 1.89 and 1.84 to 2.05 for heparin and EDTA blood DNA respectively. The second readings were taken after a gap of three months storage at -20˚C. The differences between the second and first reading for DNA extracted from heparin and EDTA blood, were estimated. The concentration of DNA from heparin and EDTA blood cells ranged from 0.1 to 3.2 (with the exception of 19.02 in one sample), and 0.1 to 1.5 respectively. Similarly the differences between OD of two readings in case of heparinised and EDTA DNA were observed more or less same as indicated in table 1. Similarly the differences between third second readings, fourth – third and fifth – fourth were also observed. The observations on concentration of genomic DNA indicate degradation of DNA extracted from heparinised blood and EDTA blood was ranged from 1.5 to 91.6 (Table 2, Fig.1) and 2.5 to 268.4 (Table 3, Fig.2) respectively during one year. The OD readings were more or less same for all the DNA samples. The degradation of EDTA DNA was rather higher than heparinised DNA.

**Table 1:** Concentration and optical density (OD) of DNA extracted from blood using different anticoagulants


	23/10/2012			23/01/2013		Difference		23/04/2013		Difference		23/07/2013		Difference		23/10/2013		Difference	
	BULL ID	Conc.	260/280	Conc.	260/280	Conc.	260/280	Conc.	260/280	Conc.	260/280	Conc.	260/280	Conc.	260/280	Conc.	260/280	Conc.	260/280

**Heparin**	558	299.9	1.87	299	1.86	0.9	0.01	234.4	1.84	64.6	0.02	234.1	1.84	0.3	0	227	1.84	7.1	0
	594	79.9	1.88	78.5	1.87	1.4	0.01	54.4	1.84	24.1	0.03	54.4	1.84	0	0	54.4	1.84	0	0
	575	432.8	1.86	432.1	1.85	0.7	0.01	369.8	1.84	62.3	0.01	369.2	1.84	0.6	0	369.2	1.84	0	0
	2456	172	1.87	171.9	1.86	0.1	0.01	128.4	1.82	43.5	0.04	128.1	1.82	0.3	0	128.1	1.82	0	0
	573	151.5	1.87	150.6	1.86	0.9	0.01	150	1.83	0.6	0.03	150	1.83	0	0	150	1.83	0	0
	557	29.3	1.86	27.4	1.85	1.9	0.01	20.8	1.82	6.6	0.03	19.2	1.8	1.6	0.02	19.2	1.8	0	0
	570	296.8	1.83	295.5	1.83	1.3	0	210.3	1.83	85.2	0	205.2	1.82	5.1	0.01	205.2	1.82	0	0
	589	265.4	1.87	262.2	1.86	3.2	0.01	223.9	1.83	38.3	0.03	204.6	1.82	19.3	0.01	204.6	1.82	0	0
	593	40.15	1.89	21.13	1.89	19.02	0	17.9	1.85	3.23	0.04	17.1	1.84	0.8	0.01	17.1	1.84	0	0
	590	350.1	1.86	349.9	1.85	0.2	0.01	288.1	1.84	61.8	0.01	288	1.83	0.1	0.01	288	1.83	0	0
**EDTA**	558	399.5	1.85	398	1.84	1.5	0.01	242.2	1.84	155.8	0	242.1	1.84	0.1	0	242.1	1.84	0	0
	594	428.5	1.86	427.6	1.85	0.9	0.01	364	1.84	63.6	0.01	364	1.84	0	0	364	1.84	0	0
	575	276.5	1.88	276	1.88	0.5	0	128	1.88	148	0	128	1.88	0	0	126.9	1.88	1.1	0
	2456	282	1.87	281.3	1.86	0.7	0.01	281	1.86	0.3	0	280.9	1.86	0.1	0	261.9	1.86	19	0
	573	262	2.05	261.8	2.05	0.2	0	250.2	2.04	11.6	0.01	250	2.02	0.2	0.02	185.3	2.02	64.7	0
	557	313.9	1.86	313.8	1.85	0.1	0.01	180.9	1.82	132.9	0.03	180.9	1.82	0	0	180.8	1.82	0.1	0
	570	513.2	1.84	512.2	1.83	1	0.01	244.8	1.83	267.4	0	244.8	1.83	0	0	244.8	1.83	0	0
	589	37.7	1.88	36.45	1.85	1.25	0.03	35.4	1.85	1.05	0	35.2	1.85	0.2	0	35.2	1.84	0	0.01
	593	853.1	1.84	853.1	1.84	0	0	731.6	1.84	121.5	0	731.2	1.84	0.4	0	731.2	1.84	0	0
	590	247.1	1.91	246.3	1.91	0.8	0	246.1	1.87	0.2	0.04	240	1.87	6.1	0	240	1.86	0	0.01


EDTA; Ethylenediaminetetraacetic acid, ID; identification number of bull and Conc.; Concentration

**Table 2:** Concentration of DNA extracted from Heparinised blood


Sample	Conc. (1)	Conc. (2)	Conc. (3)	Conc. (4)	Conc. (5)	Final difference(Conc.1-Conc.5)

**558**	299.9	299	234.4	234.1	227	72.9
**594**	79.9	78.5	54.4	54.4	54.4	25.5
**575**	432.8	432.1	369.8	369.2	369.2	63.6
**2456**	172	171.9	128.4	128.1	128.1	43.9
**573**	151.5	150.6	150	150	150	1.5
**557**	29.3	27.4	20.8	19.2	19.2	10.1
**570**	296.8	295.5	210.3	205.2	205.2	91.6
**589**	265.4	262.2	223.9	204.6	204.6	60.8
**593**	40.15	21.13	17.9	17.1	17.1	23.05
**590**	350.1	349.9	288.1	288	288	62.1


Conc.; Concentration.

**Table 3:** Concentration of DNA extracted from EDTA blood


Sample	Conc. (1)	Conc. (2)	Conc. (3)	Conc. (4)	Conc. (5)	Final difference(Conc.1-Conc.5)

**558**	399.5	398	242.2	242.1	242.1	157.4
**594**	428.5	427.6	364	364	364	64.5
**575**	276.5	276	128	128	126.9	149.6
**2456**	282	281.3	281	280.9	261.9	20.1
**573**	262	261.8	250.2	250	185.3	76.7
**557**	313.9	313.8	180.9	180.9	180.8	133.1
**570**	513.2	512.2	244.8	244.8	244.8	268.4
**589**	37.7	36.45	35.4	35.2	35.2	2.5
**593**	853.1	853.1	731.6	731.2	731.2	121.9
**590**	247.1	246.3	246.1	240	240	7.1


EDTA; Ethylenediaminetetraacetic acid and Conc.; Concentration.

**Fig.1:**
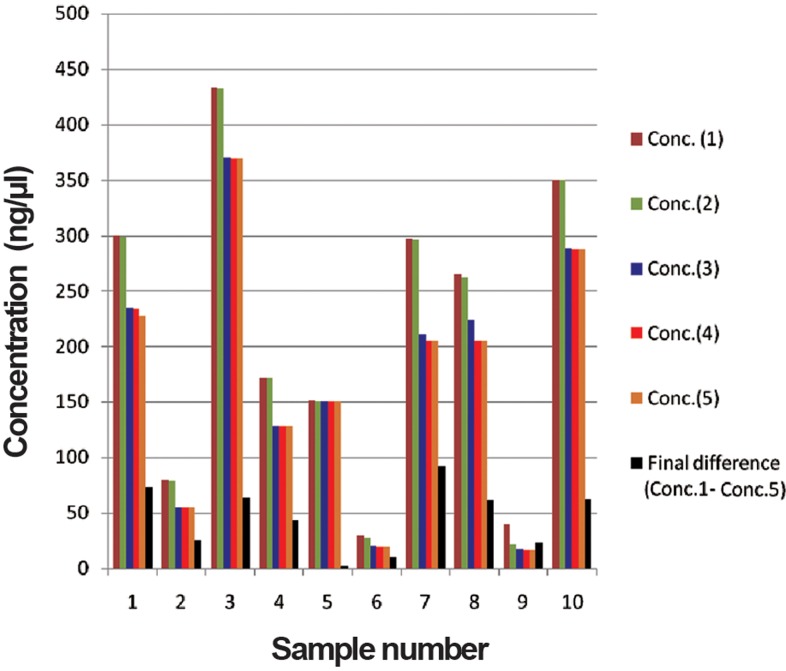
Concentration of DNA extracted from heparinised blood. Conc.; Concentration.

**Fig.2:**
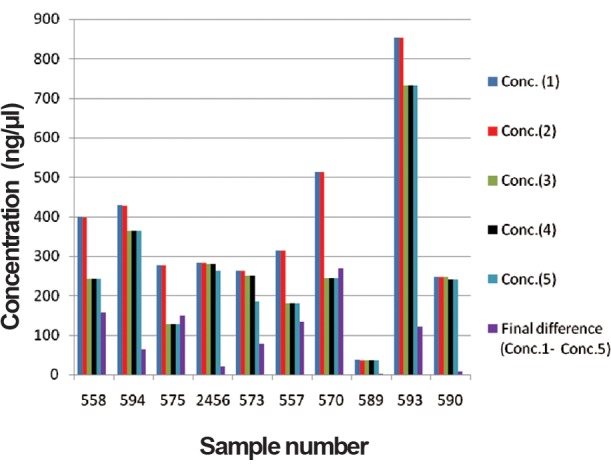
Concentration of DNA extracted from EDTA blood. Conc; Concentration and EDTA; Ethylenediaminetetraacetic acid.

After storage for a year, the quality of DNA extracted from blood using the two anticoagulants was also visualized in 0.8% agarose (Fig.3), which indicates good quality. As a test the same DNA samples were used for the following downstream application; PCR for factor XI deficiency syndrome in cattle. Gel electrophoresis on 1.5% agarose indicated quality PCR product of 244 bp (Fig.4). DNA extracted from blood samples using the two anticoagulants revealed equally good quality of PCR product for genetic disease diagnosis in cattle. There is a paucity of literatures comparing the quality and quantity of DNA extracted from blood using various anticoagulants. However, literature exists on the effect of collection, handling, transportation conditions and storage conditions before and after extraction of DNA (1, 8). Information can also be obtained from some of the pamphlets published by Norgen Biotek (www.norgenbiotek.com) and Biomerica.

The present study shows that the DNA extracted from heparin tubes can also be effectively used for genetic disease diagnosis in cattle.

**Fig.3:**
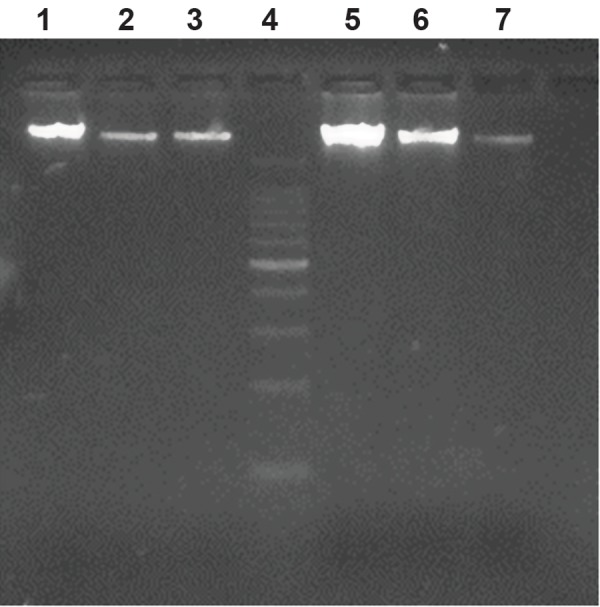
Lane 1, 2 and 3 indicate DNA extracted from blood samples collected in Heparin tubes, lane 4 is DNA ladder of 100 bp and lane 5, 6 and 7 indicate DNA extracted from EDTA blood. EDTA; Ethylenediaminetetraacetic acid.

**Fig.4:**
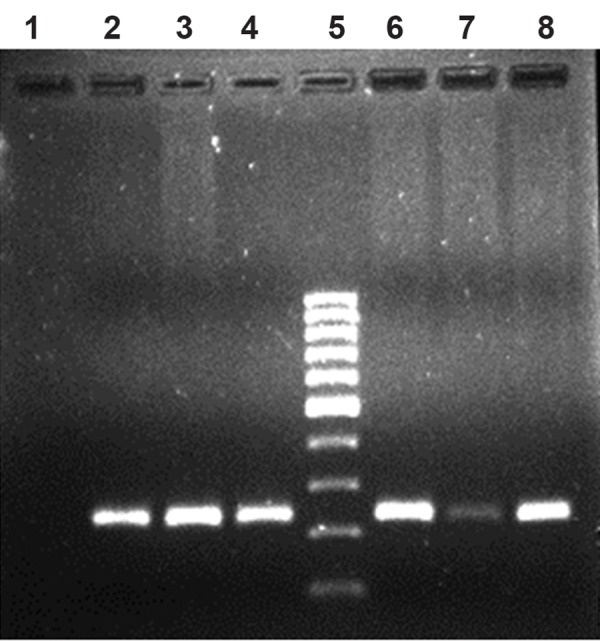
PCR product by using FXI specific primers. Lane 1 indicates blank, lane 2, 3 and 4 indicate PCR product by using DNA extracted from Heparin blood, lane 5 indicates DNA ladder of 100 bp and lane 6, 7 and 8 indicate PCR product by using DNA extracted from EDTA blood. PCR; Polymerase chain reaction, FXI; Factor XI deficiency syndrome and EDTA; Ethylenediaminetetraacetic acid.
